# Preparation and *In Vivo* Evaluation of Dichloro(1,2-Diaminocyclohexane)platinum(II)-Loaded Core Cross-Linked Polymer Micelles

**DOI:** 10.1155/2012/905796

**Published:** 2012-07-15

**Authors:** Hardeep S. Oberoi, Natalia V. Nukolova, Yi Zhao, Samuel M. Cohen, Alexander V. Kabanov, Tatiana K. Bronich

**Affiliations:** ^1^Department of Pharmaceutical Sciences and Center for Drug Delivery and Nanomedicine, College of Pharmacy, University of Nebraska Medical Center, Omaha, NE 68198-5830, USA; ^2^Department of Chemistry, M.V. Lomonosov Moscow State University, Leninskie Gory, V-234, Moscow 119992, Russia; ^3^Department of Pathology and Microbiology and Havlik-Wall Professor of Oncology, University of Nebraska Medical Center, Omaha, NE 68198-3135, USA

## Abstract

The therapeutic performance of oxaliplatin can be improved by incorporating the central *cis*-dichloro(1,2-diaminocyclohexane)platinum(II) (DACHPt) motif into the core cross-linked block copolymer micelles. We describe here the preparation, cellular uptake, and *in vivo* evaluation of core cross-linked micelles loaded with DACHPt. Stable drug-loaded micelles were prepared at high drug loading (*~*25 w/w%) and displayed a considerably increased *in vitro* cytotoxicity compared to free oxaliplatin against A2780 ovarian cancer cells. The DACHPt-loaded micelle formulation was well tolerated in mice and exhibited improved antitumor activity than oxaliplatin alone in an ovarian tumor xenograft model.

## 1. Introduction

Since the introduction of cisplatin in clinical trials in 1970's and its successful approval for the treatment of testicular cancer and a number of other malignancies, a massive research effort has been dedicated towards finding new platinum complexes with improved therapeutic efficacy. Studies on second-generation platinum complexes, designed to reduce the dose-limiting toxicities associated with cisplatin treatment, saw the successful development of carboplatin with markedly reduced incidences of renal toxicity [1]. The design of third-generation platinum complexes was intended to overcome cellular resistance to cisplatin/carboplatin. Amongst the thousands of platinum complexes designed and evaluated, *cis*-dichloro(1,2-diaminocyclohexane)platinum(II) (DACHPt), containing the DACH modification at the amine ligands of cisplatin, was recognized as a potent anticancer agent [2]. The structural modification greatly altered the activity and toxicity of this platinum complex yielding a much broader spectrum of activity and more importantly a lack of cross-resistance with cisplatin. The issue of limited DACHPt solubility was dealt with oxalate modification at the leaving groups yielding oxaliplatin which gained worldwide clinical approval [3].

Although treatment with the third-generation platinum complex-oxaliplatin is relatively better tolerated than previous generation cisplatin and carboplatin treatment, various side effects still limit its effectiveness [4]. Neurotoxicity is the most severe toxicity associated with oxaliplatin regime and is observed clinically at two stages. A transient acute syndrome appears shortly after the first few infusions and causes distal and perioral paresthesias/dysesthesias, muscular cramps and spasms [5]. The more detrimental dose limiting cumulative sensory neuropathy develops gradually and results in persisting dysesthesias and paresthesias of the extremities, impaired sensory ataxia, and deficits in fine sensory motor coordination which may impair normal life [6]. Oxaliplatin is considered to be the least nephrotoxic amongst the platinum drugs in clinical use [6]. However, there have been several reported cases of renal failure with repeated cycles of oxaliplatin administration [7–9]. In particular, patients with moderate renal impairment are at greater risk of developing cumulative renal damage with oxaliplatin treatment [10]. It is important to note that the biological activity of oxaliplatin is mediated by the DACHPt motif since the oxalate group is displaced by water and other nucleophiles, such as, chloride ions upon systemic administration [11]. There is some evidence of involvement of these oxalate ions in oxaliplatin-mediated toxicities [12].

The therapeutic performance of oxaliplatin can be enhanced by incorporating the central DACHPt motif into a macromolecular carrier [13–15]. Increased tumor suppression and reduced toxicity have been reported in preclinical and early clinical studies, attributed primarily to the long circulating behavior and enhanced permeability and retention (EPR)-mediated tumor accumulation of such carriers. The purpose of this study is to evaluate a recent class of high-capacity drug carriers, the block copolymer micelles with cross-linked ionic cores (*cl*-micelles) for delivery of DACHPt. Capable of environmentally responsive swelling, these *cl*-micelles behave as nanoscale ionic gels and can incorporate drug molecules at capacities exceeding 30 wt.%. This is significantly higher than what has been reported for liposomes and other colloidal carriers [10]. Incorporation of cisplatin into these micelles by polymer-metal complex formation was shown to improve cisplatin pharmacokinetics, enhance its antitumor efficacy, and eliminate cisplatin-mediated nephrotoxicity in a mouse model of ovarian cancer [16] and thus motivated us to explore their potential in delivery of the more potent DACHPt analog. We describe the preparation, cellular uptake and *in vivo* evaluation of *cl-*micelles loaded with DACHPt (DACHPt/*cl*-micelles). Although a behavior similar to cisplatin-micelles was expected due to the presence of identical leaving groups and geometry of the central Pt atom in both these Pt-analogs, our results indicate a considerably different cytotoxicity, cellular DNA platination, and *in vivo* toxicity for DACHPt/*cl*-micelles. This suggests a strong influence of the drug cargo on the effectiveness of *cl*-micelles and indicates that a potent drug-carrier combination would need to be evaluated on a case-by-case basis.

## 2. Materials and Methods

### 2.1. Materials

Poly(ethylene oxide)-*b*-poly(methacrylic acid) (PEO-*b*-PMA) diblock copolymer (Mw/Mn = 1.45, block lengths 170 units PEO and 180 units PMA) was from Polymer Source Inc., Canada. The concentration of carboxylate groups in the copolymer samples was estimated by potentiometric titration. Oxaliplatin was from LC Laboratories (Woburn, MA, USA). DACHPt (dichloro(1,2-diaminocyclohexane)platinum(II)), silver nitrate (AgNO_3_), 1,2-ethylenediamine (ED), 1-(3-dimethylaminopropyl)-3-ethylcarbodiimide hydrochloride (EDC), and *α*-D-glucose (dextrose) were from Sigma–Aldrich (St Louis, MO, USA). Platinum (Pt) standard for inductively coupled plasma-mass spectrometer (ICP-MS) calibration was from Acros Organics (Geel, Belgium). Iridium chloride internal standard for ICP-MS was from Spex Certiprep (Metuchen, NJ). Double distilled nitric acid, 70%, and 6 M hydrochloric acid were from GFS Chemicals (Columbus, OH, USA). Heparinized tubes for blood collection (Microtainer tubes) were from BD (Franklin Lakes, NJ, USA).

### 2.2. General Procedure for the Synthesis of *cl*-micelles

The general procedure for synthesis of cross-linked PEO-*b*-PMA micelles has been described previously [17]. Briefly, PEO-*b*-PMA/Ca^2+^ complexes were prepared by mixing an aqueous solution of PEO-*b*-PMA with a solution of CaCl_2_ at a molar ratio of [Ca^2+^]/[COO^−^] = 1.3. Chains were cross-linked overnight at room temperature (r.t.) using ED and EDC. Extent of cross-linking was targeted to 20% and was controlled by the ratio of amine functional groups of ED to carboxylic acid groups of polymer chains. After completion of reaction, a 1.5 molar excess amount of EDTA relative to [Ca^2+^] was added and followed with extensive dialysis, first against 0.5% aqueous ammonia and then against distilled water to remove Ca^2+^ ions and by-products of cross-linking reaction.

### 2.3. Preparation of DACHPt/*cl*-micelles

DACHPt (5 mM) was suspended in distilled water and mixed with silver nitrate ([AgNO_3_]/[DACHPt] = 1) in the dark at r.t. for 24 h to form the aqueous complex. AgCl precipitates formed after reaction were removed by centrifugation at 3000 rpm for 10 min followed by filtration through a 0.22 *μ*m filter. The aqueous dispersions of *cl*-micelles were mixed with the aqueous solution of DACHPt (~1 mg/mL) at pH 7.0 at a 0.5 molar ratio of DACHPt to carboxylate groups of the *cl-*micelle followed by incubation at 25°C for 48 h. Unbound DACHPt was removed by ultrafiltration using Centricon Plus-20 centrifugal filter units (MWCO 30,000 Da, Millipore, Billerica, MA, USA). Drug content was measured by Pt (Pt194/Pt195) assay on an ICP-MS (NexION 300Q, PerkinElmer, Waltham, MA, USA) calibrated with Pt (2 to 100 ng/mL).

### 2.4. Drug Release Studies

Drug release from *cl*-micelles in phosphate-buffered saline (PBS, pH 7.4, 0.14 M NaCl) or acetate buffer with saline (ABS, pH 5.5, 0.14 M NaCl) was determined using Spectra/Por Float-A-Lyzer G2 dialysis systems (MWCO 3.5–5.0 kDa, Spectrum Laboratories, Rancho Dominguez, CA, USA). Pt content in dialysate was measured using ICP-MS and expressed as percent of total versus time.

### 2.5. Particle Characterization

Particles were characterized by dynamic light scattering (DLS), zeta (*ζ*)-potential, effective hydrodynamic diameters (*D*_eff_), polydispersity indices (PDI) and atomic force microscopy (AFM) as described previously [18].

### 2.6. Cell Culture and Cytotoxicity Assay

A2780 human ovarian cancer cells were cultured in RPMI 1640 medium (RPMI) with 10% (v/v) FBS, 100 U/mL penicillin, and 0.1 mg/mL streptomycin at 37°C, 5% CO_2_. For the cytotoxicity assay, cells were seeded in 96-well plates (5000 cells/well) 24 h before the experiment, treated with free oxaliplatin, DACHPt/*cl*-micelles (0 to 300 *μ*g drug/mL) or empty *cl*-micelles for 24, 48, or 72 h, and then cultured for additional 24 h in drug-free media at 37°C. Cell survival was assessed by a standard colorimetric MTT assay as described previously [19]. IC_50_ values were calculated using GraphPad Prism 5 software package (GraphPad Software, La Jolla, CA, USA).

### 2.7. Intracellular Pt(II) Accumulation

 A2780 cells were seeded in duplicate 25 cm^2^ flasks, allowed to reach 80–90% confluence, and treated with free oxaliplatin or DACHPt/*cl*-micelles for 24 h (37°C) at DACHPt equivalent concentrations of 1.0, 2.5, and 10.0 *μ*M. Following incubation, each flask was rinsed three times with PBS, trypsinized, aliquoted in duplicate 1.5 mL microcentrifuge tubes and pelleted by centrifugation. One of each duplicate cell pellets was wet ashed by overnight incubation in 70% nitric acid at 60°C and analyzed for Pt(II) content by ICP-MS. The other duplicate was lysed using M-PER mammalian protein extraction reagent (Thermo Scientific, Franklin, MA, USA) and quantified for total protein using a micro BCA protein assay kit (Pierce, Rockford, IL, USA) as per the manufacturer's instructions. Whole cell uptake was expressed as ng Pt/mg cellular protein.

### 2.8. DNA Platination

 Duplicate 25 cm^2^ flasks containing A2780 cells (80–90% confluence) were treated with free oxaliplatin or DACHPt/*cl*-micelles for 24 h (37°C) at a DACHPt equivalent concentration of 1.0, 2.5, and 10.0 *μ*M. Following incubation, each flask was rinsed three times with PBS, trypsinized, and aliquoted in 1.5 mL microfuge tubes and pelleted by centrifugation. Cellular DNA was isolated using Qiagen DNeasy tissue kit (Qiagen, Valencia, CA, USA) as per the manufacturer's instructions. Upon determination of the DNA concentration, the remaining solution was wet ashed in 70% nitric acid at 60°C and analyzed for Pt(II) content by ICP-MS. DNA platination was expressed as ng Pt/*μ*g DNA.

### 2.9. Animal Facility and Standard of Care

Upon arrival, animals were placed in an AAALAC-accredited facility. Food and reverse osmosis (RO) water were available ad libitum throughout the study. Euthanasia was performed by CO_2_ asphyxiation. Treatments were administered by tail vein injection. Drug amount was calculated based on the average animal body weight. All animal protocols were approved by the UNMC Institutional Animal Care and Use Committee (IACUC).

### 2.10. Maximum Tolerated Dose and *In Vivo* Toxicity

Six-week-old female C57Bl/6 mice were purchased from Charles River Breeding Laboratories (Raleigh, NC, USA), quarantined for 7 days, and randomized based on body weight into treatment groups (*n* = 3). Treatment was administered by bolus i.v. tail vein injection, 4 treatments in total, with each treatment every fourth day. The treatment groups comprised free oxaliplatin at doses of 2 mg, 4 mg, 6 mg, and 8 mg oxaliplatin/kg body weight, DACHPt/*cl*-micelles at doses of 2 mg, 4 mg, 6 mg, and 8 mg DACHPt/kg body weight, and 5% dextrose solution as control. All treatments were given in an identical treatment regimen. Animals were monitored daily for stress, and body weights of all animals were measured every second day during the course of the study.

At the end of the study on day 13, animals were sacrificed and blood, kidneys, liver, and spleen were collected. Kidneys, liver, and spleen were weighed and fixed in buffered formalin. Blood was collected in heparinized tubes and analyzed for plasma concentrations of blood urea nitrogen (BUN), creatinine, aspartate aminotransferase (AST), and alanine aminotransferase (ALT) at the Nebraska Medical Center clinical laboratory using a UniCel DxC 600 Synchron Clinical System (Beckman, CA, USA). Fixed tissues were processed, sectioned, inserted into tissue cassettes, dehydrated in 70% ethanol overnight, and paraffin embedded (UNMC Tissue Sciences Facility). Serial 5 *μ*m sections were stained with hematoxylin and eosin (H&E). For histopathological diagnosis, H&E-stained slides were examined by light microscopy and photomicrographs were taken using a Nikon camera mounted on a Nikon Eclipse 600 microscope (Melville, NY, USA) with Adobe Elements 3.0 software (Adobe Systems, Mountain View, CA, USA).

### 2.11. Antitumor Activity

Antitumor activity was evaluated in female nude mice (Nu-/nu-, National Cancer Institute, Bethesda, MD, USA) bearing subcutaneous A2780 cell ovarian xenografts. Xenografts were initiated by injecting A2780 cells (5 × 10^6^ cells/site) subcutaneously into the flanks, one above each hind limb. When tumor size was approximately 100 mm^3^ (10–12 days after inoculation), animals were randomized based on tumor volume into 3 treatment groups with 8 animals per group. Treatment was administered by bolus i.v. tail vein injection, 4 treatments in total, with each treatment every fourth day. The treatment groups comprised free oxaliplatin at a dose of 4 mg oxaliplatin/kg body weight, DACHPt/*cl*-micelles at a dose of 4 mg DACHPt/kg body weight, and 5% dextrose solution. All treatments were given in a volume of 100 *μ*L per injection in an identical treatment regimen. The animal body weight and tumor volume were monitored every second day. Tumor volume was estimated by measuring two orthogonal diameters (longer dimension: *L*, and smaller dimension: *W*) of the tumor using electronic calipers. The tumor volume was defined as *V* = 0.5 × *L* × *W* ^2^. The animals were sacrificed when the tumor volume exceeded 3000 mm^3^, greatest tumor dimension exceeded 20 mm, the tumor became necrotic, or the animal exhibited a body weight loss of more than 20%. All other animals were sacrificed by day 20, which was considered the experimental endpoint. 

## 3. Results

PEG-*b*-PMA *cl*-micelles represent hydrophilic nanogel-like structures with a swollen core of cross-linked PMA network surrounded by a PEG shell. Their synthesis, characterization, and ability to incorporate anticancer drugs, cisplatin and doxorubicin, were described earlier [17, 18].

### 3.1. Preparation of DACHPt/*cl*-micelles

**Figure 1:**
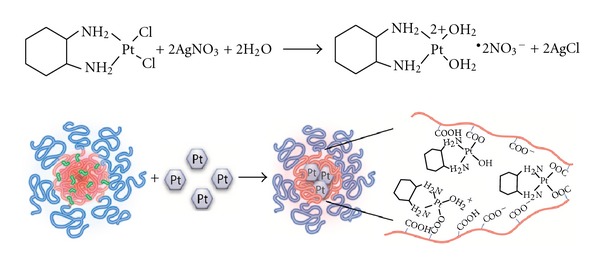
Scheme of the preparation of DACHPt/*cl*-micelles. DACHPt is first reacted with silver nitrate, forming the platinum complexes with much greater water solubility, followed by immobilization into *cl*-micelles by simple mixing in salt-free conditions.

**Figure 2:**
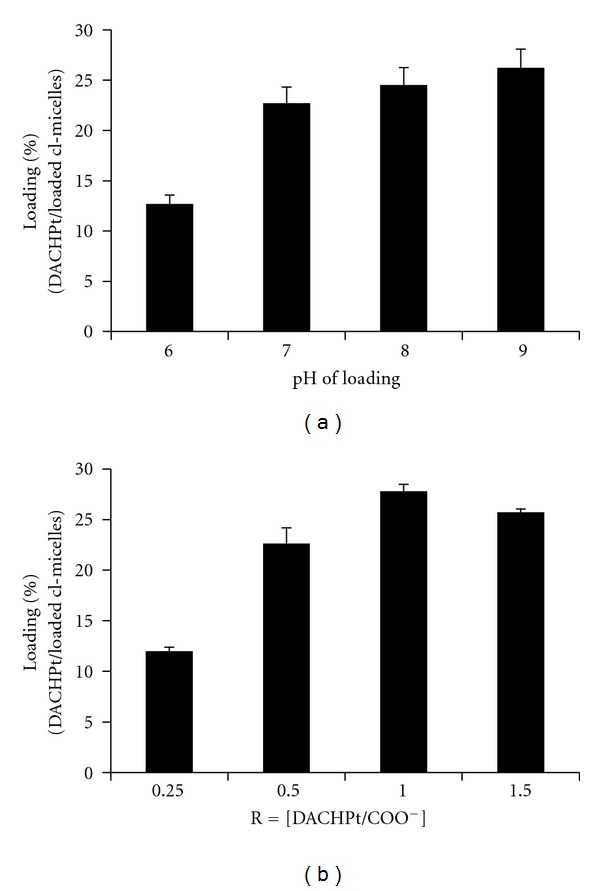
Preparation of DACHPt/*cl*-micelles. (a) Effect of pH on drug-loading capacity of *cl*-micelles. (b) Effect of varying molar ratios of DACHPt to carboxylate groups of *cl-*micelles (R) on drug loading. Values are mean ± standard deviation (*n* = 2).

 The aqueous solubility of DACHPt is very low (~0.25 mg/mL). Therefore, DACHPt was first reacted with silver nitrate, forming the platinum complexes with much greater water solubility, then immobilized into *cl*-micelles by simple mixing at room temperature (Figure 1). Drug binding in the micelle core occurs presumably by polymer-metal complex formation. Initially, the formulation conditions (pH of mixture during drug loading and molar ratio of drug to carboxylate groups of PEO-*b*-PMA) were optimized.  Figure 2(a) shows the effect of pH on DACHPt-loading capacity of *cl*-micelles: platinum incorporation into micelles increased from ~12% at pH 6.0 to ~23% at pH 7.0, above which there was only a marginal increase in drug loading. Formulations prepared at pH > 7.0 were not stable and resulted in formation of precipitates within 2 days. Therefore, pH 7.0 was chosen as the optimal pH value for drug loading. Further, the molar ratio of DACHPt to carboxylate groups of *cl-*micelles (*R* = [DACHPt]/[COO^−^]) was varied at this optimal pH from ca. 0.25 to 1.5. Above *R* = 0.5 there was no appreciable increase in drug loading (Figure 2(b)) while a significant decrease in loading efficiency (percentage of drug incorporated into the *cl*-micelles for a given amount of drug added to the mixture) was observed. The *R* = 0.5 was hence chosen as the optimal ratio for DACHPt loading. The drug-loaded *cl*-micelles prepared as such were stable in dispersion and did not exhibit any aggregation for more than 30 days at room temperature.

### 3.2. Characterization of DACHPt/*cl*-micelles

 The size, *ζ*-potential, and composition of *cl*-micelles were determined by a combination of physicochemical methods (Table 1). The DACHPt-loaded and empty *cl*-micelles had a size below 200 nm and a net negative *ζ*-potential. The topology of the *cl*-micelles was further studied by AFM. Both empty (Figure S1A, see Supplementary Materials available online at doi:10.1155/2012/905796) and DACHPt/*cl*-micelles (Figure S1B) appeared to be spherical particles. The average height and diameter of particles is shown in Table S1. Drug incorporation proceeded with a marginal decrease in size and *ζ*-potential (Table 1) as determined by DLS. Similar trend was evident in AFM images as an overall decrease in both height and diameter of the *cl-*micelles (Table S1). This is consistent with the neutralization of the PMA chains upon DACHPt binding to carboxylate groups, resulting in reduction of the osmotic swelling pressure inside the cores of *cl*-micelles. Despite high drug loading (~25 wt.%), DACHPt/*cl*-micelles retained a negative *ζ*-potential, suggesting that not all available carboxylate groups were consumed upon drug binding. There is a possibility that some of the DACHPt is physically entrapped and retained in the *cl-*micelles due to the nonspecific hydrophobic interactions of the cyclohexane group of DACHPt with DACHPt/PMA complex regions.

### 3.3. Release of DACHPt from *cl*-Micelles

**Figure 3:**
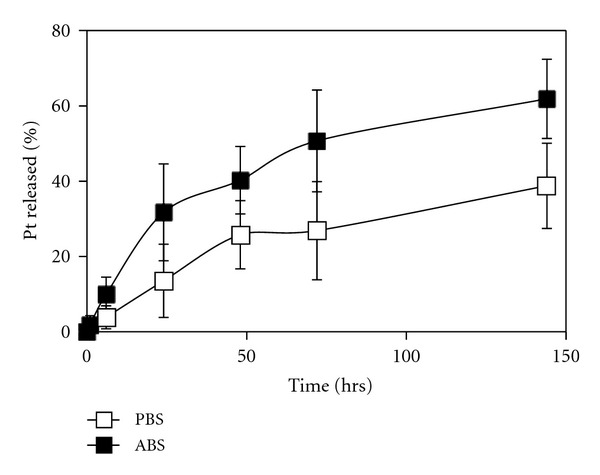
Drug release profile for DACHPt/*cl*-micelles. Release of DACHPt in PBS (□) or ABS (■) at 37°C. Values are mean ± standard deviation (*n* = 3).

 The release behavior of Pt(II) complexes from *cl*-micelles was studied by equilibrium dialysis, in either phosphate buffered saline (PBS, pH 7.4) or acetate buffer saline (ABS, pH 5.5) at 37°C (Figure 3). The conditions reflect the pH encountered in plasma and intracellular compartments, respectively. The drug-carrier interaction was essentially reversible with a slow and sustained drug release profile and lack of any burst release. The release is assumed to be largely mediated by ligand exchange reactions between the carboxylate groups of PMA and the chloride, phosphate, or acetate ions in the release media. The accelerated release at the acidic pH may be due to protonation of carboxylic groups of PMA, which would weaken the drug-polymer interaction. The observed release behavior could allow accelerated drug release in hypoxic tumors and intracellular compartments characterized by an acidic environment.

### 3.4. Cellular Uptake, DNA Platination and *In Vitro* Cytotoxicity

**Figure 4:**
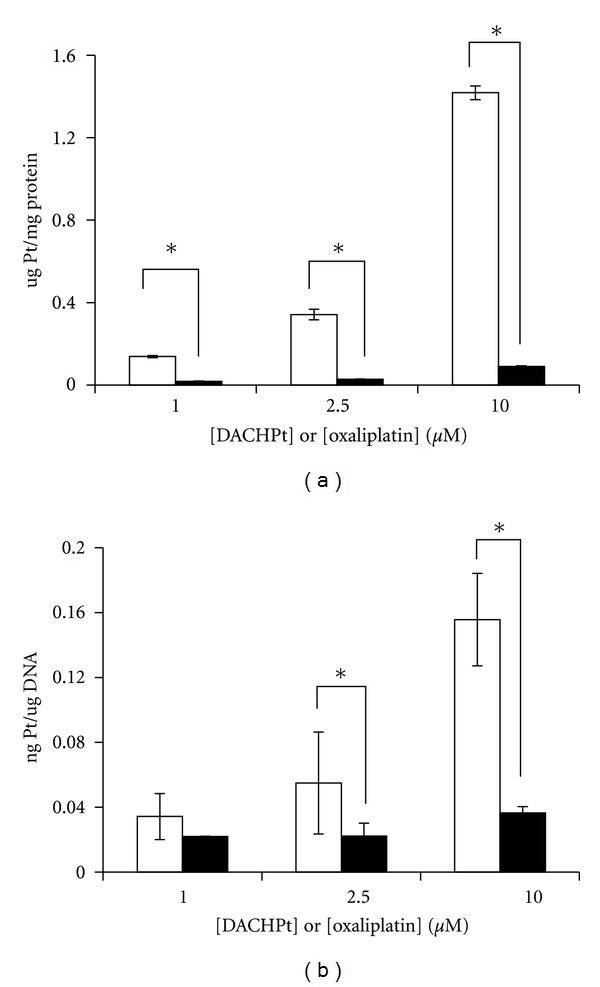
Cellular uptake and DNA platination.(a) Whole-cell Pt accumulation and (b) extent of DNA platination, following incubation with 1.0, 2.5, and 10 *μ*M oxaliplatin (filled bars) or equivalent doses of DACHPt/*cl*-micelles (open bars) following 24 h exposure in A2780 cells as measured by ICP-MS. Values are mean ± standard deviation (*n* = 3).

 Cellular accumulation of platinum is a key step in cellular platinum drug pharmacology. To examine relative accumulation of DACHPt/*cl*-micelles in cancer cells, the total Pt(II) content was measured in A2780 cells following their exposure to oxaliplatin or DACHPt/*cl*-micelles at varying drug concentrations. The data, normalized to the total cellular protein, are presented in Figure 4(a). The net cellular accumulation of DACHPt/*cl*-micelles was substantially higher compared to that of the free drug. The increased intracellular uptake of Pt(II) with the DACHPt/*cl*-micelles also translated into an increase in the amount of drug that reached its primary biological target, the DNA. Analysis of the extent of nucleus DNA platination measured after 24 h exposure of A2780 cells to various concentrations of DACHPt/*cl*-micelles or free oxaliplatin, shown in Figure 4(b), indicates higher DNA-platination at all concentrations of DACHPt/*cl*-micelles tested. It is generally accepted that DNA damage induced by binding of platinum drugs is largely responsible for their cytotoxic properties. Thus, the higher DNA platination not surprisingly manifested as an increased cytotoxicity of DACHPt/*cl*-micelles compared to the free oxaliplatin against A2780 human ovarian cancer cell line. The IC_50_ values for each formulation are summarized in Table 2. The cytotoxic activity of DACHPt/*cl*-micelles was solely mediated by the released drug as empty polymer micelles did not affect cell survival (shown previously, [17]). The cytotoxicity data further provides evidence of the reversible nature of drug interaction with the carrier, with release of biologically active Pt(II) complexes under physiological conditions.

### 3.5. Maximum Tolerated Dose and Toxicity in C57Bl/6 Mice

**Figure 5:**
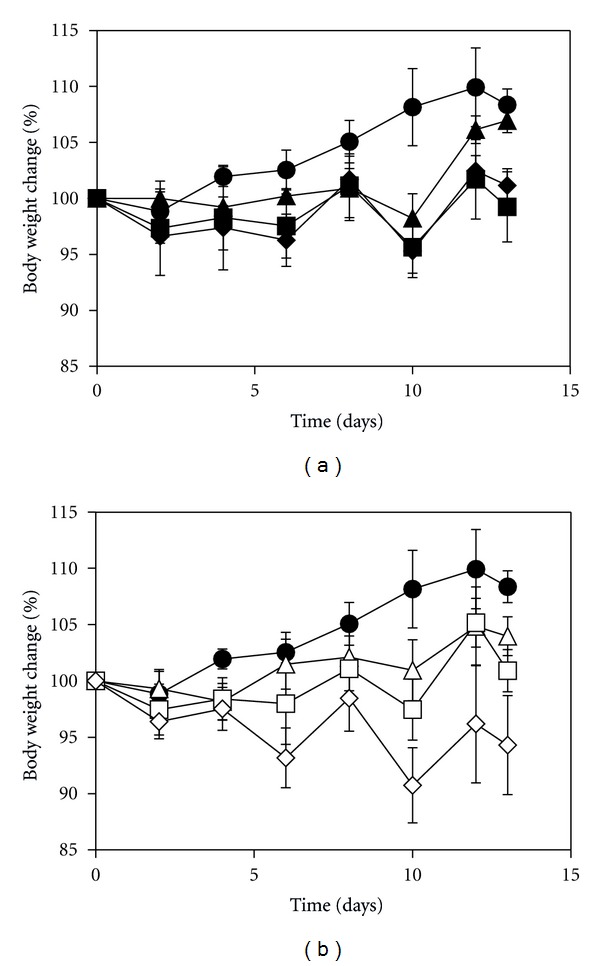
Relative changes in body weight in C57Bl/6 mice. (a) Oxaliplatin was injected i.v. every fourth day, four administrations in total, at a dose of (▲) 2 mg, (■) 4 mg, and (*◆*) 6 mg oxaliplatin/kg body weight. (b) DACHPt/*cl*-micelles were injected at identical treatment schedule at a dose of (∆) 2 mg, (□) 4 mg, and (*◊*) 6 mg DACHPt/kg body weight. Dextrose 5% treatment (*⚫*) was used as the control. Values are mean ± standard deviation (*n* = 3).

**Figure 6:**
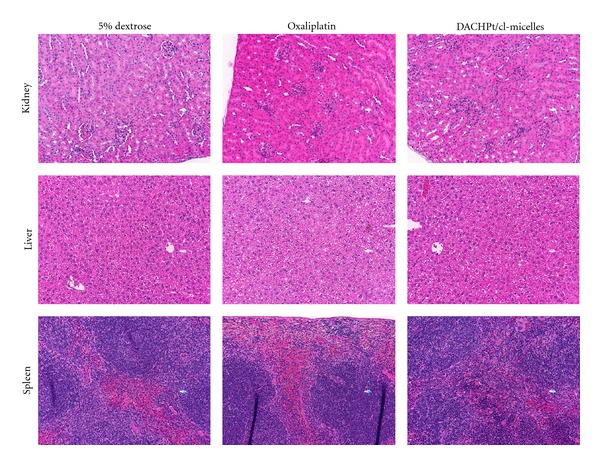
Light microscopy images of H&E stained kidney, liver, and spleen. Images were taken at a magnification of 200x. Tissue samples were collected at day 13 following i.v. administration of oxaliplatin or DACHPt/*cl*-micelles at 4 mg/kg body weight. Vehicle control groups received 100 *μ*L of 5% dextrose. Four administrations were given in total with each administration at every fourth day.

 The maximum tolerated dose (MTD) of free oxaliplatin and DACHPt/*cl*-micelles was evaluated in female C57Bl/6 mice following i.v. bolus administration of 2 mg, 4 mg, 6 mg, and 8 mg drug equivalents/kg body weight every fourth day with 4 treatments in total. Figure 5 depicts the relative changes in body weight. Greater than 20% decreased body weight gain was observed in the animals treated at 8 mg drug equivalents of oxaliplatin and DACHPt/*cl*-micelles (data not shown), indicating significant toxicity. All other treatment groups had less than 10% decreased body weight gain and hence did not reveal any major toxicity. DACHPt/*cl*-micelle treatment group at 6 mg/kg dose had a slightly more decreased weight gain compared to free oxaliplatin treatment group (~10% decreased wt. gain in DACHPt/*cl*-micelles group compared to ~5% in oxaliplatin group at day 10). Plasma BUN, creatinine, alkaline phosphatase, AST, and ALT concentrations were measured at sacrifice (Table 3). Although animals in the free oxaliplatin treatment group had a reduced serum creatinine, and elevated ALT and AST levels were detected in DACHPt/*cl*-micelles treatment group compared to controls, the values were within the normal range reported in the literature [20]. Tissue weight analysis at sacrifice (Table 4) indicated significant differences in kidney to body weight ratios between oxaliplatin treatment groups at 4 mg and 6 mg oxaliplatin/kg body weight compared to controls. No such difference was seen in kidney tissues of the animals treated with DACHPt formulations. There was a significant change in liver/body weight ratio in 4 mg DACHPt/*cl*-micelle treatment group. However, light microscopic examination of H&E stained tissue sections did not indicate any significant histopathological changes in any of the treatment groups at the MTD (Figure 6) or lower treatment doses (Figures S2 and S3). In contrast, one surviving animals which received treatment at 8 mg DACHPt/*cl*-micelles (higher than MTD) exhibited pronounced pathological changes in liver tissue characterized by inflammation and necrosis.

Altogether, no major signs of toxicity were observed up to 6 mg drug/kg body weight dose in any of the groups. Oxaliplatin treatment only had an acceptable decreased body weight gain of less than 10% but had a reduced kidney to body weight ratio. DACHPt/*cl*-micelle treatment group also had a decreased body weight gain less than 10% but indicated increased liver to body weight ratio, surprisingly at 4 mg drug/kg compared to 6 mg drug/kg. However, considering the widely known dose dependent nature of platinum-drug toxicity, 4 mg drug/kg dose was chosen as the MTD for further studies.

### 3.6. Antitumor Efficacy of DACHPt/*cl*-micelles in Mice

**Figure 7:**
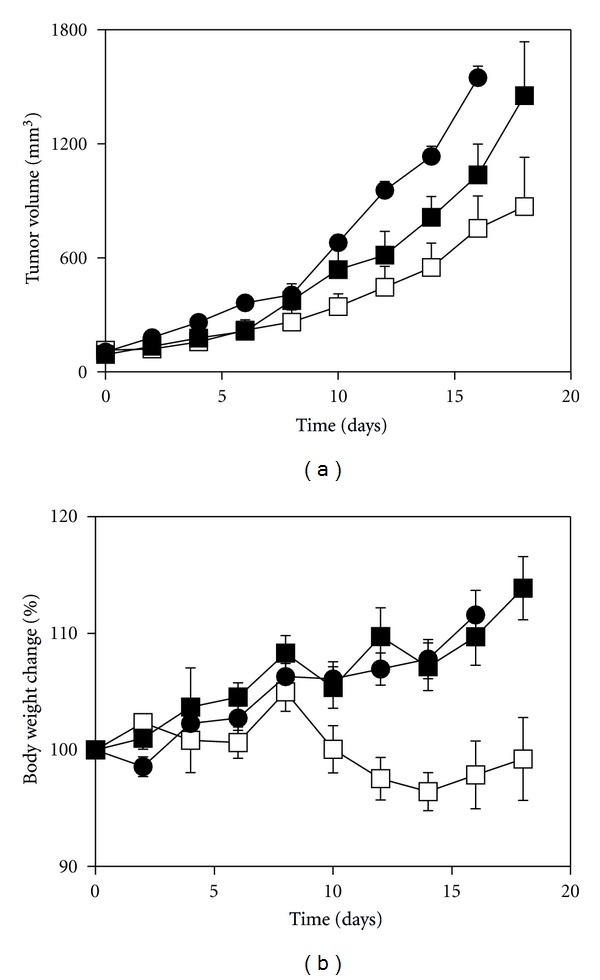
*In vivo* efficacy of DACHPt/*cl*-micelles in A2780 human ovarian cancer xenograft-bearing female nude mice. Relative changes in (a) tumor volume and (b) body weight were measured following i.v. administration of (□) DACHPt/*cl*-micelles or (■) free oxaliplatin at 4 mg drug equivalents/kg body weight. Four administrations were given in total with each administration at every fourth day. Control group received 100 *μ*L of 5% dextrose. Values are mean ± SEM (*n* = 8).

The antitumor activity of drug-loaded *cl-*micelles was evaluated in mice bearing A2780 human ovarian cancer xenografts. Oxaliplatin or DACHPt/*cl*-micelles were administered at 4 mg drug equivalents/kg body weight every fourth day, 4 treatments in total. Changes in relative tumor volume and body weight are shown in Figures 7(a) and 7(b), respectively. Both the oxaliplatin and DACHPt/*cl*-micelle treatments significantly decreased the tumor growth rates compared to the control group (Figure 7(a)). Tumor burden in the animals treated with DACHPt/*cl*-micelles was the least and significantly smaller (*P* < 0.05) compared to free oxaliplatin treatment group. Although the body weight loss in DACHPt/*cl*-micelle treatment groups was significantly different from the control and oxaliplatin treatment groups, animals did not show any major signs of stress and had a body weight change less than 10% until the termination of study.

## 4. Discussion

 Carrier-based delivery of anticancer drugs has received much attention in recent years for its potential to improve drug efficacy and reduce unwanted side effects. The carriers designed to “selectively target cancer” exploit differences between normal and cancerous tissues as biological targets, primarily through their enhanced permeability and retention (EPR effect) [21]. Amongst the various nanomaterials being explored, block copolymer micelles with cross-linked ionic cores represent interesting materials well suited for delivery of platinum complexes. Platinum drugs are somewhat soluble in aqueous media and are not readily incorporated into nanoparticles with a hydrophobic interior. Polymer micelles with “ionic cores” allow platinum drug incorporation by utilizing the ability of transition metal platinum to form a coordination complex with the carboxylates at the polymer backbone. This polymer-metal complex formation permits exceptional drug loading capacity, c.a. ~30% for cisplatin and ~25% for DACHPt reported here. Moreover, the carboxylic groups of the copolymer involved in Pt-drug complexation have a low nucleophilicity, which permits release of the platinum drug at physiologic salt concentrations. A precise control over the macroscopic characteristics of *cl-*micelle via block copolymer chemical composition, total molecular weight and block length ratios [22], and the ease of drug loading by simple mixing are some of the other unique advantages with this system.

 We had previously reported the preparation of cisplatin-loaded *cl-*micelles and demonstrated improved pharmacokinetics, enhanced antitumor efficacy, and elimination of cisplatin-mediated nephrotoxicity in a mouse model of ovarian cancer [16]. *cl*-Micelles incorporating the more potent DACHPt analog exhibited some interesting differences from the previously described cisplatin-loaded *cl-*micelles that indicate a strong influence of the drug cargo on the effectiveness of *cl*-micelles. There were distinct differences in the formulation aspects with regards to the incorporated drug. While stable cisplatin-loaded *cl*-micelles can be prepared over a broad range of pH at their maximal loading capacity, DACHPt/*cl*-micelles prepared above pH 7 exhibited lower dispersion stability and precipitated over time. It is widely known that the colloidal stability of such drug-loaded micelles is critically dependent upon the ability of the hydrophilic block to minimize the interfacial free energy and impede its interaction with other particles. Therefore, it appears that the PEO segments in the corona of *cl-*micelles were unable to stabilize the particles in the dispersion above a certain content of loaded DACHPt because of the relatively higher hydrophobicity of the DACHPt/PMA cores compared to cisplatin/PMA core. The optimal molar ratio of drug to carboxylate groups of PEO-*b*-PMA copolymer used for drug loading was similar for both DACHPt and cisplatin. However, the changes in size and *ζ*-potential of *cl*-micelles following drug incorporation differed significantly. At relatively comparable loading capacities of *cl*-micelles, cisplatin loading resulted in a much greater decrease of particle size and increase in *ζ*-potential compared to DACHPt loading suggesting that less carboxylate groups were consumed upon DACHPt binding. It is reasonable to assume that the steric constraints imposed by bulky DACH moieties may alter drug binding through coordination interactions with carboxylate groups of the PMA chains. It appears that DACHPt incorporation into the *cl-*micelles involves additional mechanisms different from polymer-metal complex formation. But further investigations are needed to confirm this hypothesis. Despite the observed differences, the drug release behavior was similar for both formulations.

 It is worth noting that DACHPt/*cl*-micelles exhibited more potent cytotoxicity relative to free oxaliplatin (Table 2). This is in sharp contrast to cisplatin-loaded *cl-*micelles and most other nanoparticulate formulations of Pt-based drugs, which typically show reduced cytotoxicity relative to the corresponding free drug. Such differences in behavior can, at least in part, be attributed to the differences in the cellular uptake and subcellular transport of Pt-containing species. Indeed, exposure of A2780 cells to DACHPt/*cl*-micelles resulted in significantly higher accumulation of platinum than did exposure to oxaliplatin (Figure 4(a)). This is probably because *cl*-micelles are able to efficiently internalize and deliver its cargo in the cancer cells via endocytosis [23], while oxaliplatin enters the cells either by passive diffusion or using organic cation/copper transporters [24]. The amount of platinum bound to cellular DNA was also higher for the DACHPt formulation compared with oxaliplatin at equimolar concentrations, correlating with the extent of the intracellular platinum accumulation. It is interesting to note here that while a similar increased cellular accumulation was observed for cisplatin-loaded *cl-*micelles described previously [25], the net cellular DNA platination was significantly reduced after exposure to micellar cisplatin compared to the free drug. Considering that the *in vitro* drug release profiles for DACHPt- or cisplatin-loaded *cl-*micelles are not much different, differences in endosomal/lysosomal escape of the respective drugs appears to be the determining process affecting their cytotoxicity. Faster endosomal escape has previously been reported for hydrophobic peptides [26], and their conjugates [27], and therefore, the greater hydrophobicity of DACHPt relative to cisplatin are likely to be a contributing factor to the observed behavior.

Toxicity analysis in C57Bl/6 mice did not reveal any major toxicity from the treatment with DACHPt formulations up to a dose of 6 mg DACHPt/kg body weight. These treatment groups had a body weight loss less than 10% and a normal tissue histopathology. Some aberration in ALT/AST levels and liver/body weight ratio compared to controls was seen in one of the DACHPt/*cl*-micelle treatment group (4 mg/kg). Although these values remained within the normal range reported in the literature [20], they could be indicative of an early stage subclinical toxicity. While biodistribution experiments were not performed in this study, it is reasonable to expect the elevated accumulation of DACHPt-micellar formulations in the liver and spleen, as has been seen with cisplatin-loaded *cl-*micelles [16]. Considering the higher potency of DACHPt in general compared to cisplatin as well as the ability of DACHPt/*cl*-micelles to deliver substantially higher dose of cytotoxic Pt species into the cells, the high drug accumulation in these tissues could be the cause of the observed changes in blood chemistry. Significant changes in serum creatinine and kidney/body weight ratios were also seen in oxaliplatin treatment groups, but again they were within the normal range defined in literature. Decreased serum creatinine is generally associated with muscle wasting [28], which is not a major toxicity associated with oxaliplatin treatment [6]. A long-term evaluation of toxicity markers is needed to ascertain if these observed changes in oxaliplatin and DACHPt/*cl*-micelles treatment groups indeed translate into any significant toxicity.

The antitumor activity of drug-loaded *cl*-micelles evaluated in mice bearing A2780 human ovarian cancer xenografts indicated significantly decreased tumor growth rates compared to the control and oxaliplatin treatment groups. It is difficult to ascertain whether DACHPt/*cl*-micelles with the more active DACHPt analog had antitumor activity higher than that with cisplatin-loaded *cl-*micelles [16]. Such a comparison could not be performed across the different experimental setups due to the inherent variability in animal experiments and marked differences in tumor growth rates as such. A head-to-head comparison in the same experimental setup would have been ideal but was not performed in this study.

## 5. Conclusions

 Hydrophilic polymer micelles with cross-linked ionic cores were evaluated as potential drug carriers for oxaliplatin parent complex, DACHPt. High drug loading (up to 25 w/w%) was achieved with slow and sustained pH-dependent release of platinum species in physiological solutions. DACHPt/*cl*-micelles exhibited considerably higher *in vitro* cytotoxicity compared to oxaliplatin in A2780 ovarian cell cultures, which was mechanistically traced to increased Pt cellular accumulation and DNA platination. Toxicity analysis in animals revealed no major toxicity, although some elevation in liver and spleen enzymes ALT and AST was observed. DACHPt/*cl*-micelles were significantly more effective in inhibiting the growth of ovarian subcutaneous tumors than oxaliplatin at the MTD level. These results suggest that the *cl-*micelle drug delivery system may be useful for improving the efficacy of platinum chemotherapy in ovarian cancer.

## Supplementary Material

The supplementary material section contains tapping mode AFM images of empty and DACHPt-loaded *cl*-micelles, light microscopy images of H&E stained kidney, liver and spleen tissues from animals treated with 2 mg/kg and 6 mg/kg DACHPt-loaded *cl*-micelles and a tabular description of dimensions of DACHPt-loaded *cl*-micelles analyzed by AFM.

## Figures and Tables

**Table 1:** Physicochemical properties of DACHPt/*cl-*micelles^a^.

	*D*_eff_ (nm)	PDI^b^	*ζ*-potential (mV)	LC^c^ (%)	LE^d^ (%)
Empty *cl*-micelles	170 ± 20	0.12	−38 ± 8	n.a	n.a
DACHPt/*cl-*micelles	154 ± 10	0.10	−30 ± 5	25	45

^a^Values determined at pH 7.0, values are mean ± standard deviation.

^b^PDI: polydispersity index.

^c^LC: loading capacity (percentage of drug by weight incorporated into drug loaded *cl-*micelles), *R* = 0.5.

^d^LE: loading efficiency (percentage of drug incorporated into *cl-*micelles for a given amount of drug added to the mixture), *R* = 0.5.

**Table 2:** *In vitro* cytotoxicity of free oxaliplatin and DACHPt/*cl-*micelles against A2780 ovarian cancer cell line.

Treatment	IC_50_ (DACHPt equivalents in *μ*g/mL)^a^	
	Oxaliplatin	DACHPt/*cl-*micelles
24 h	0.982 ± 0.05	0.812 ± 0.11
48 h	0.726 ± 0.07	0.433 ± 0.09^b^
72 h	0.517 ± 0.15	0.114 ± 0.07^b^

^a^Values are mean ± standard deviation (*n* = 3).

^b^Significantly different from oxaliplatin treatment (*P* < 0.05).

**Table 3:** Clinical chemistry parameters from female C57Bl/6 mice^a^.

Treatment	ALT	AST	BUN	Creatinine
	(U/L)	(U/L)	(mg/dL)	(mg/dL)
Normal range^b^	17–77		9–33	0.1–0.4
Control	26 ± 2.6	83 ± 8	13.3 ± 2.1	0.250 ± 0.053

		Oxaliplatin		

2 mg/kg	29 ± 1.4	82 ± 27.6	14.7 ± 4	**0.160 ± 0.017** ^**c**^
4 mg/kg	32 ± 2.5	**110 ± 47.5** ^**c**^	13.0 ± 1.0	**0.157 ± 0.015** ^**c**^
6 mg/kg	30 ± 1.0	88 ± 13.1	12.0 ± 1.7	**0.160 ± 0.010** ^**b**^

	DACHPt/*cl-*micelles			

2 mg/kg	**45 ± 2.1** ^**c**^	**149 ± 21.9** ^**c**^	12.0 ± 2.0	0.230 ± 0.046
4 mg/kg	**63 ± 3.0** ^**c**^	**181 ± 36.9** ^**c**^	8.7 ± 0.6	0.203 ± 0.021
6 mg/kg	43 ± 29.0	143 ± 51.6	10.7 ± 2.9	0.170 ± 0.044

^a^Values are mean ± standard deviation (*n* = 3).

^b^As reported by Roy et al. [20]

^c^Significantly different from the controls (*P* < 0.05).

**Table 4:** Tissue weight analysis at sacrifice from female C57Bl/6 mice^a^.

Treatment	Liver/BW	Spleen/BW	Kidney/BW
Control	0.0448 ± 0.0039	0.0041 ± 0.0013	0.0142 ± 0.0007

	Oxaliplatin		

2 mg/kg	0.0418 ± 0.0013	0.0028 ± 0.0007	0.0131 ± 0.0006
4 mg/kg	0.0469 ± 0.0021	0.0021 ± 0.0003	**0.0126 ± 0.0007** ^**b**^
6 mg/kg	0.0459 ± 0.0025	0.0022 ± 0.0000	**0.0130 ± 0.0004** ^**b**^

	DACHPt/*cl-*micelles		

2 mg/kg	0.0471 ± 0.0041	0.0037 ± 0.0005	0.0140 ± 0.0007
4 mg/kg	**0.0542 ± 0.0025** ^**b**^	0.0040 ± 0.0007	0.0153 ± 0.0003
6 mg/kg	0.0461 ± 0.0048	0.0038 ± 0.0005	0.0152 ± 0.0004

^a^Values are mean ± standard deviation (*n* = 3).

^b^Significantly different from the controls (*P* < 0.05).
