# Supplementary material for: Preparation and In Vivo Evaluation of Dichloro(1,2-Diaminocyclohexane)platinum(II)-Loaded Core Cross-Linked Polymer Micelles
Source: Chemother Res Pract. 2012 Jul 15;2012:905796. doi: 10.1155/2012/905796 (PMC3403332; doi:10.1155/2012/905796)
Supplement: Supplementary file 1 — The supplementary material section contains tapping mode AFM images of empty and DACHPt-loaded cl-micelles, light microscopy images of H&E stained kidney, liver and spleen tissues from animals treated with 2 mg/kg and 6 mg/kg DACHPt-loaded cl-micelles and a tabular description of dimensions of DACHPt-loaded cl-micelles analyzed by AFM. [file 905796.f1.pdf]

Supplementary Information

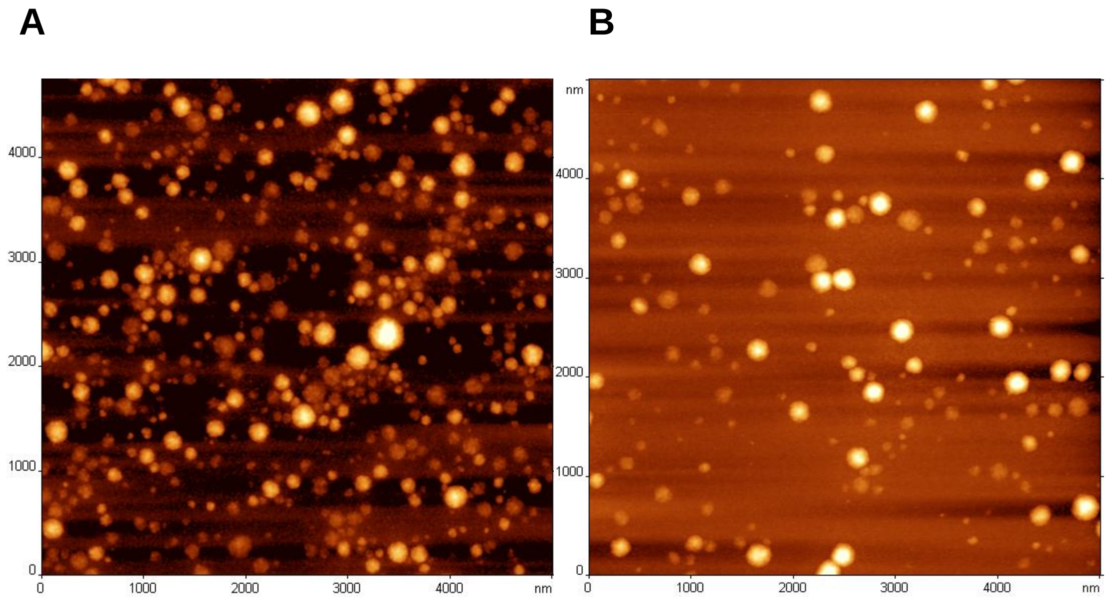

Figure S1: Tapping mode AFM images of *cl*-micelles in air, (A) empty micelles and (B) DACHPt-loaded micelles. The samples were prepared by depositing 5 µl of an aqueous dispersion of nanogels (1 mg/ml) onto 1-(3-aminopropyl)silatrane-modified mica surface (APS-mica) for 5 min followed by surface drying under argon. AFM imaging was performed in air using a Multimode NanoScope IV system (Veeco, Santa Barbara, CA, USA) operated in a tapping mode and regular etched silicon probes (TESP) with a spring constant of 42 N/m. The particles mean volume, height and width (not corrected for convolution effect) were determined using Femtoscan Online software (Advanced Technologies Center, Moscow, Russia).

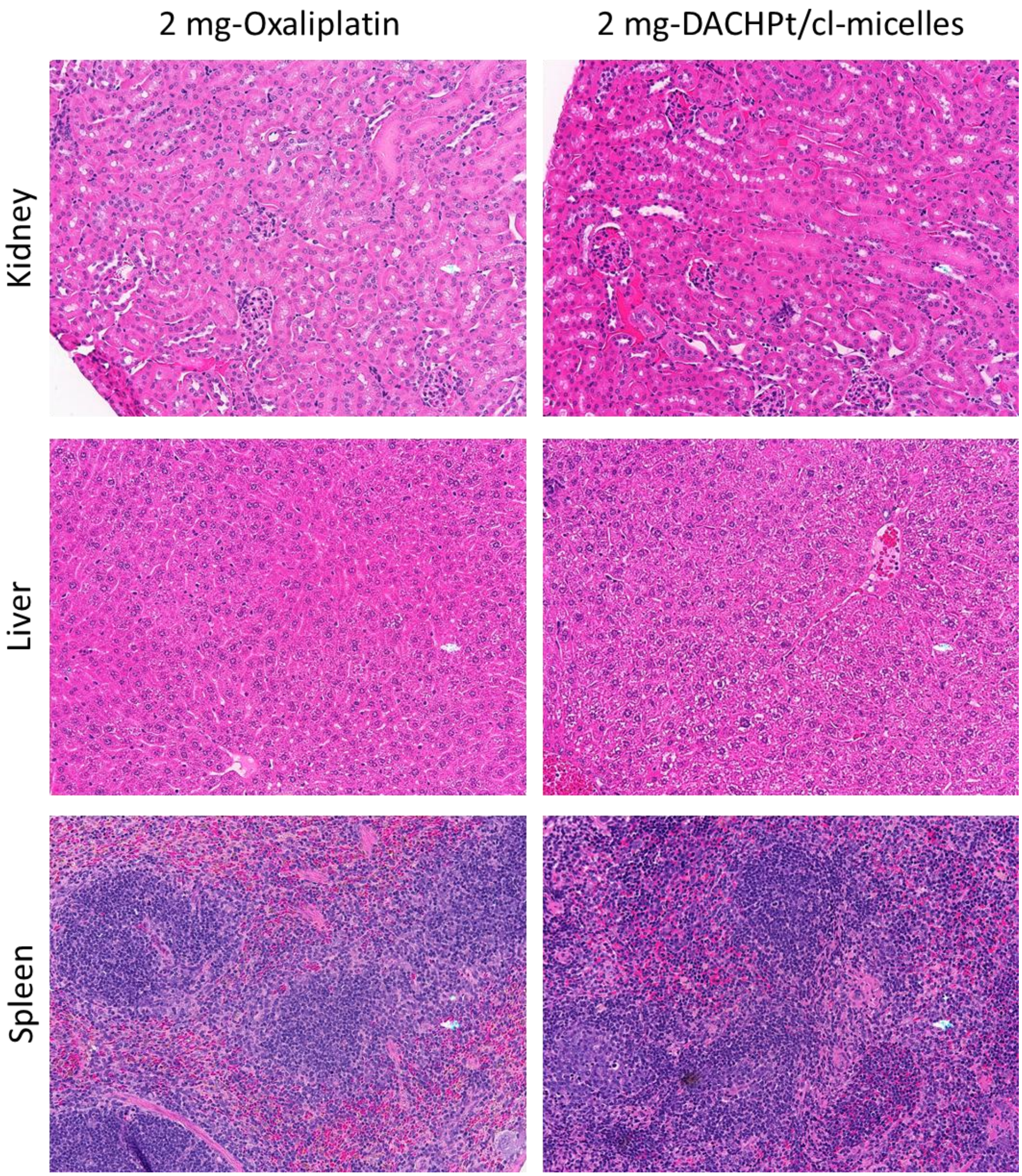

Figure S2: Light microscopy images of H&E stained kidney, liver and spleen tissues from animals with 2 mg/kg treatment administration (200x). Vehicle control groups received 100 µl of 5% dextrose. Four administrations were given in total with each administration at every fourth-day. Tissue samples were collected at day 13.

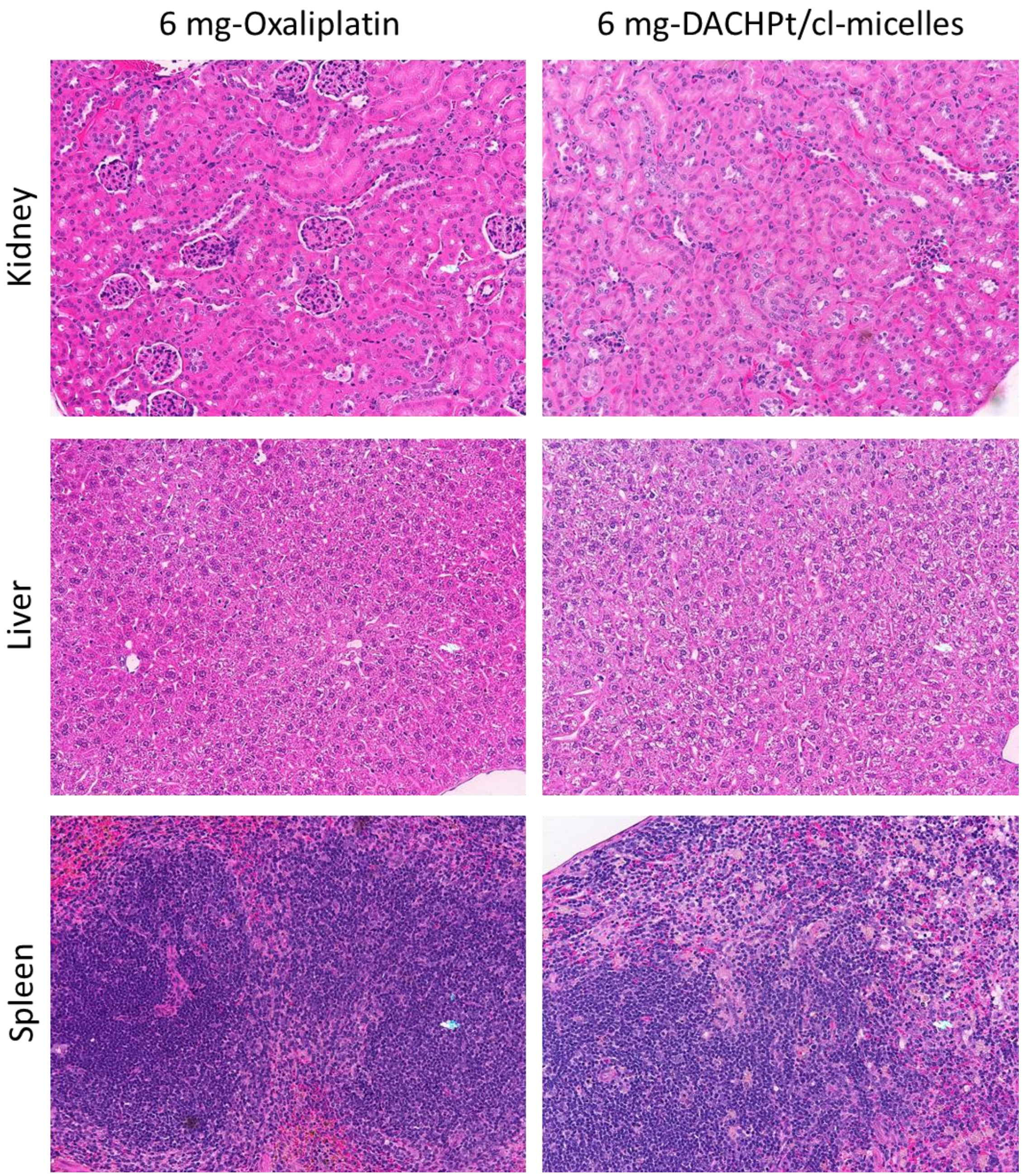

Figure S3: Light microscopy images of H&E stained kidney, liver and spleen tissues from animals with 6 mg/kg treatment administration (200x). Vehicle control groups received 100 µl of 5% dextrose. Four administrations were given in total with each administration at every fourth-day. Tissue samples were collected at day 13.

Table S1: Dimensions of DACHPt-loaded *cl*-micelles analyzed by AFM

	H_{av} (nm)[a]	D_{av} (nm)[b]
Empty cl-micelles	6.17 ± 1.14	96.07 ± 57.6
DACHPt loaded micelles	2.88 ± 0.84	91.29 ± 59.9

[a]Number-averaged heights (H_{av}) of the micelles.

[b] Number-averaged diameters (D_{av}) of the micelles.
